# Cultivation Optimization and Structural Characterization of *Stephanocyclus meneghinianus* for Sustainable High-Quality Biosilica Production

**DOI:** 10.3390/nano15130971

**Published:** 2025-06-22

**Authors:** Daeryul Kwon, Yoseph Seo, Chaehong Park, Sang Deuk Lee, Taek Lee

**Affiliations:** 1 Protist Research Division, Biological Resources Research Department, Nakdonggang National Institute of Biological Resources (NNIBR), 137, Donam 2-gil, Sangju-si 37182, Republic of Korea; diatom83@nnibr.re.kr; 2 Department of Chemical Engineering, Kwangwoon University, 20 Kwangwoon-ro, Nowon-gu, Seoul 01897, Republic of Korea; akdldytpq12@kw.ac.kr; 3 Encountter the Ecology, Gwanggyojungang-ro 248, Yeongtonggu, Suwon 16512, Republic of Korea; qkrcoghd2@gmail.com

**Keywords:** freshwater, diatom, *Stephanocyclus meneghinianus*, structure, biosilica

## Abstract

This study investigates the potential use of the freshwater centric diatom *Stephanocyclus meneghinianus* as a sustainable source of high-purity biosilica. We analyzed its morphology, microstructure, and optimal culture conditions, and developed a pretreatment method to recover intact biosilica frustules. The isolated diatoms exhibited small and uniform cell sizes (8–10 μm) with distinctive features such as regularly arranged spines, striae, and fultoportulae. Electron microscopy revealed around 4000 nanoscale pores per valve, mostly along the costae. The pretreatment process using ethanol and hydrogen peroxide effectively removed organic materials and mucilage, preserving the structural integrity of the biosilica. Crystallinity analysis confirmed the amorphous nature of the biosilica, indicating good biodegradability, while elemental analysis showed its composition as being primarily of silicon and oxygen. Growth optimization experiments revealed the highest specific growth rate in DM medium at 20–25 °C under light intensities of 60–120 μmol m^−2^ s^−1^. These results demonstrate that *S. meneghinianus* can be cultured efficiently to produce biodegradable biosilica with well-defined nanostructures. This biosilica shows promise for applications in biomaterials, nanotechnology, pharmaceuticals, and environmental remediation.

## 1. Introduction

Diatoms are essential components of aquatic ecosystems worldwide and represent one of the most remarkable and ecologically significant groups of microalgae [1,2]. These organisms inhabit diverse aquatic environments, ranging from marine to freshwater systems, and are major contributors to global carbon fixation [3,4]. Diatoms are estimated to account for up to 20% of the oxygen produced annually on Earth, rivaling the contribution of terrestrial forests to the planet’s oxygen levels [2,5]. Diatoms are unicellular microalgae characterized by their silica-based cell walls, which are naturally produced and uniquely structured [6,7]. What makes diatoms exceptional is their biologically intricate cell wall, known as the frustule, composed of silica (SiO_2_) [6,7,8]. The frustules are not only structurally complex but also highly organized at the nanoscale, making diatoms an outstanding example of natural nanotechnology [9,10]. Diatom-based natural biosilica is environmentally friendly, biocompatible, and biodegradable, making it an ideal candidate for diverse applications in medicine, cosmetics, and nanomaterials [11,12]. In particular, the biocompatibility of biosilica opens up potential applications in drug delivery systems, tissue engineering, and cosmetic formulations [12,13]. Furthermore, the high surface area, porosity, and mechanical strength of biosilica make it highly useful in catalysis, environmental remediation, and advanced optical material development [14]. Consequently, research into leveraging diatoms as sustainable sources of biosilica has garnered significant attention [13,15]. Among various diatom species, the round and highly porous *Stephanocyclus meneghinianus* (formerly *Cyclotella meneghiniana*) has emerged as a particularly promising candidate for biosilica-related studies due to its well-defined morphology and favorable structural properties [12,15]. This species is commonly found in freshwater environments and exhibits unique structural features and growth adaptability [16]. The frustules of *S. meneghinianus* display distinct morphological characteristics, including highly porous and complex hierarchical patterns [17]. These nanoscale features make this species particularly interesting for exploring the production and application of biosilica [18]. Efficient production of biosilica is closely linked to the optimization of growth conditions for diatoms [19]. Factors such as light intensity, temperature, pH, and nutrient availability significantly influence both the quantity and quality of biosilica produced by diatoms [20,21]. While existing studies have provided valuable insights into specific growth conditions, to maximize biosilica production, comprehensive studies that systematically evaluate and optimize the interaction of multiple factors are essential [20,21]. Understanding the ultrastructure of diatom biosilica is equally critical for both fundamental and applied research [22]. Advanced imaging techniques such as scanning electron microscopy (SEM) and transmission electron microscopy (TEM) enable detailed visualization of the nanoscale features of diatom frustules [10,22]. These analyses are indispensable for characterizing structural integrity, surface properties, and porosity [23]. Moreover, ultrastructural studies provide critical data for developing nanostructure-based applications, such as drug delivery systems, catalytic materials, and optical devices [12,24]. This study aims to address the research gap by focusing on *S*. *meneghinianus*, a freshwater diatom species with significant potential for biosilica production and application. The primary objectives are (1) to determine the optimal culture and medium conditions for growth, (2) to analyze the micro- and ultrastructural characteristics of the frustules with an emphasis on porosity, and (3) to develop effective methods for recovering and harvesting biosilica. Through these objectives, the study seeks to establish a foundation for sustainable biosilica production and utilization, addressing both scientific and practical imperatives. By optimizing growth conditions, elucidating ultrastructural characteristics, and proposing efficient recovery methods, this research contributes to the advancement of environmentally friendly and high-value materials derived from diatoms.

## 2. Materials and Methods

### 2.1. Sampling Sites and Methods

**Figure 1:**
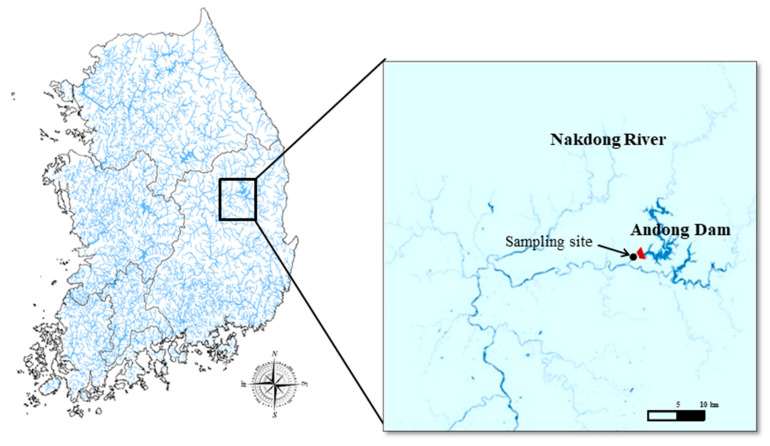
Sampling site for collection of *S. meneghinianus*.

In order to separate *S*. *meneghinianus* from freshwater diatom, collection must first be performed. Since this study aims to explore the possibility of utilizing biosilica, the collection site was selected for the Nakdonggang River’s upper water system, where there is little inflow of pollutants in Korea. Specifically, water samples were collected the headwater of the Nakdong River (Figure 1). In addition, basic water quality parameters, including water temperature, pH, salinity, electrical conductivity, dissolved oxygen (DO), and turbidity, were measured on-site using a portable water quality measuring device (ProDDS, YSI, Yellow Springs, OH, USA) to understand the environmental conditions of the collection site (Table 1). After the samples were transported to the laboratory, the presence of diatoms was confirmed using a microscope. Once diatoms were identified, individual diatom cells were isolated using a micropipette. Each isolated single cell was inoculated into a 96-well plate containing culture medium. As cell proliferation progressed, sequential subculturing was performed by transferring the cells into progressively larger wells, including 48-well, 24-well, 12-well, and 6-well plates, to adapt the cells to larger culture environments. Finally, the diatom cultures grown from single cells were transferred to cell culture flasks for continuous subculturing. Through this process, *S. meneghinianus* cultures were successfully established.

### 2.2. Analysis of Shape and Structure Through Microscope

The finally isolated and cultured strains were observed under a light microscope at magnifications ranging from 40× to 400× to examine the morphology of the diatoms. Samples were thoroughly dried to ensure stability during analysis. To enhance surface conductivity, the sample surfaces were coated with a thin layer of gold or platinum using a sputter coater under vacuum conditions. This coating process ensured uniform coverage of the sample surface. The coated samples were mounted on a field emission scanning electron microscope (Fe-SEM, MIR-3, Tescan, Brno-Kohoutovice, Czech Republic) sample holder and analyzed using a scanning electron microscope. The microscope was operated at an appropriate accelerating voltage to capture detailed surface microstructures. High-resolution images of the sample’s surface were obtained during SEM analysis. The images were digitally captured, and all observations were recorded, with scale bars to provide reference measurements. The ultrastructural analysis of *S. meneghinianus* biosilica was performed using a transmission electron microscope to characterize its nanostructural features. Initially, the diatom cultures were harvested by centrifugation at 3000 rpm for 10 min to collect the biomass. The collected biomass was washed three times with distilled water to remove any residual medium or impurities. The cleaned biosilica was dispersed in distilled water and sonicated for 10 min to ensure even dispersion of the particles. A small drop of the suspension was placed onto a copper TEM grid coated with a carbon film and allowed to dry under ambient conditions. TEM imaging was conducted using a transmission electron microscope (TEM, JEM-2100F, Tokyo, Japan) operated at an accelerating voltage of [insert voltage, e.g., 200 kV]. High resolution images were obtained to examine the detailed nanostructure, including pore size, porosity, and hierarchical organization of the frustules.

### 2.3. Analysis of Surface Elements and Crystal Structure

A scanning electron microscope (SEM) and energy dispersive X-ray spectroscopy (EDX) were used to analyze the elemental composition of the sample. The sample was thoroughly dried and then coated with a thin layer of platinum to ensure conductivity. The coating was applied to a specific thickness using a sputter coater prior to SEM analysis. Elemental analysis was performed using an EDX detector (EDX; Oxford Instruments, Abingdon, United Kingdom, 80 mm^2^ X-MaxN Silicon Drift Detector) attached to the SEM system. Both point analysis, to determine the elemental composition at specific locations, and area analysis, to examine broader regions, were conducted. Additionally, elemental mapping and line scanning were performed to visualize the spatial distribution of elements within the sample. To analyze the crystal structure and diffraction patterns of the sample, Fourier Transform (FFT) image analysis was conducted using transmission electron microscopy (TEM). After observing the ultra-fine structure of *S. meneghinianus*, the acquired TEM images were digitally processed using FFT to analyze the regolith pattern.

### 2.4. Processing Protocol for S. meneghinianus Biosilica Extraction

**Figure 2:**
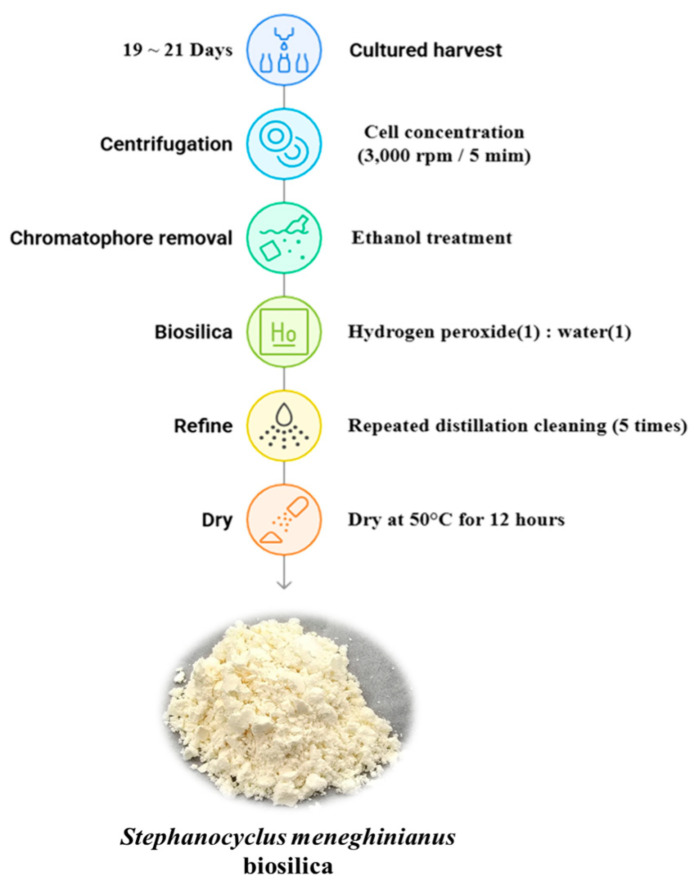
Purification and pretreatment process for obtaining single cell biosilica from *S. meneghinianus*.

The culture of *S. meneghinianus* was maintained by subculturing during the period of highest cell density, between days 19 and 21, to ensure consistent growth. During the peak growth phase, single-cell cultures of *S. meneghinianus* were harvested by centrifugation at 3000 rpm for 5 min. The supernatant was discarded, and the concentrated cells were subjected to chloroplast removal by incubation in 95% ethyl alcohol (Samchun Pure Chemical, Pyeongtaek, Republic of Korea) for 24 h. Subsequently, a mixture of 35% hydrogen peroxide (H_2_O_2_, Junsei) and distilled water (1:1, *v*/*v*) were added, and the suspension was left to stand for 24 h to perform a pretreatment process, separating the epitheca and hypotheca of *S. meneghinianus*. For purification, the pretreated *S. meneghinianus* cells were centrifuged at 3000 rpm for 5 min, the supernatant was removed, and the pellet was resuspended in distilled water. This washing process was repeated 5 to 6 times to remove residual reagents and impurities. The purified *S. meneghinianus* frustules were then dried at 50 °C for 12 h. The final biosilica materials were obtained as isolated frustules of *S. meneghinianus*, as shown in Figure 2.

### 2.5. Growth Rate

Considering the morphology and ecological characteristics of *S. meneghinianus*, we established optimal growth rate experiments and medium concentration conditions under various parameters, including temperature, light, and medium concentration (Table 2). To secure a substantial amount of biosilica, we analyzed the growth rate. Subsequently, we experimented with various culture media commonly used for freshwater diatoms and ultimately determined the optimal growth conditions concerning temperature and photoperiod for *S. meneghinianus* in the most effective medium. In the growth rate experiment, a constant density of *S. meneghinianus* was inoculated into a flask at the beginning of the experiment, and cell density was measured at 3-day intervals. The specific growth rate was calculated using the cell growth rate equation described in [25].

$$µ({day}^{-1})=ln\;(N_{1}/N_{0})/(t_{1}-t_{0})$$

*N*_1_: cell concentration (cells mL^−1^) at time t_1_. N_0_: cell concentration at time t_0_.

## 3. Results

### 3.1. Morphology and Structure of S. meneghinianus Before Preprocessing

**Figure 3:**
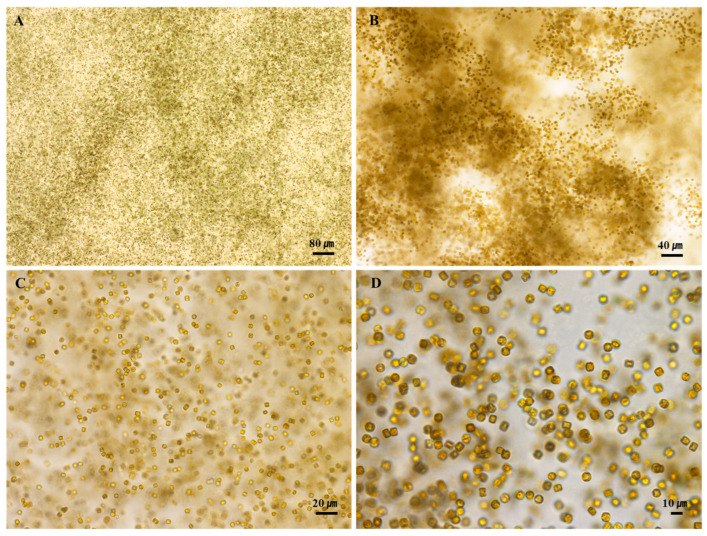
Optical microscopy image of cultured *S. meneghinianus* ((**A**) ×40; (**B**) ×100; (**C**) ×200; (**D**) ×400). (Scale bar: (**A**) = 80 µm; (**B**) = 40 µm; (**C**) = 20 µm; (**D**) = 10 µm).

**Figure 4:**
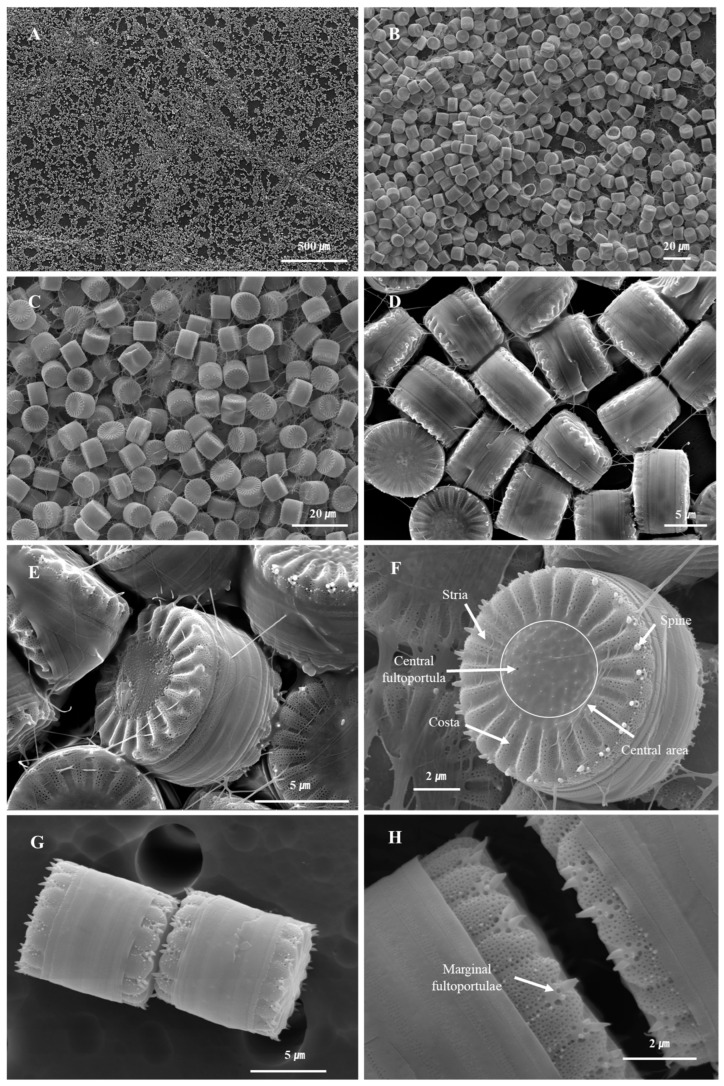
Microscopic structures of cultured *S. meneghinianus* observed by electron microscopy. (**A**–**C**) Overall morphology of cultured *S. meneghinianus*. (**D**,**E**) Lateral view showing secreted mucilage. (**F**) Detailed morphology and structure of the valve. (**G**,**H**) Gridle bands view of the valve. (Scale bar: (**A**) = 500 µm; (**B**,**C**) = 20 µm; (**D**,**E**,**G**) = 5 µm; (**F**,**H**) = 2 µm).

At the time of the investigation, the water temperature was 12.7 °C, there was 10.13 mg/L of dissolved oxygen, and the pH was 7.79, which showed an aqueous environment rich in dissolved oxygen and neutral pH in a low-temperature environment (Table 1). When the cells of *S. meneghinianus* were observed through an optical microscope, they showed a dark brown color and were found to be round, single cells (Figure 3). When observed through an electron microscope, the diameter of *S. meneghinianus* are generally known to be 6–18 μm [26,27], but the diameter of *S. meneghinianus* isolated and cultured in this study were relatively small and uniform, ranging from 8 to 10 μm (Figure 4). The upper surface of the valve is round and it can be seen that the visible protrusions, called spines, are regularly arranged around the valve (Figure 4F–H). It is a structure that combines the epitheca and the hypotheca of the valves, and the shape seen when viewed from the top is round, but when viewed from the side, it can be seen that the epitheca and the hypotheca are relatively strong and delicately combined in a cylindrical shape (Figure 4G,H). The central area is clearly defined and separated from the marginal chambered striae, occupying approximately one-third to one-half of the valve face. On the side view of the valve, a wavy horizontal pattern can be observed, where the recessed areas are referred to as striae and the raised areas as costae (Figure 4). Most of the pores are distributed along these elevated costae (Figure 4F–H). A single cell typically possesses 20 to 22 costae, each containing nanoscale pores known as areolae, which are generally circular or oval in shape (Figure 4). A single cell, including both the epitheca and hypotheca, contains approximately 4000 such pores. A fultoportula is located within the central area, and under scanning electron microscopy (SEM), marginal fultoportulae are positioned along each costa (Figure 4D–H). Research on diatom-derived nanofibrous mucilage has been conducted since the late 1970s [28,29]. These nanofibers primarily function to facilitate attachment to surfaces such as stones and plants, protect cells from external environmental stresses, and enhance the absorption of organic matter and nutrients in the surrounding environment as a survival mechanism [30,31]. However, not all diatoms produce mucilage; it is typically observed in attached genera such as *Gomphonema*, *Cymbella*, and *Encyonema* [32]. These species possess an apical pore field, observable under electron microscopy, which serves as the secretion site for mucilage [33].

In contrast, centric diatoms such as *Stephanocyclus* and *Cyclotella* species secrete nanofibrous β-chitin through both marginal fultoportulae and a single central fultoportula. These structures are involved in the extrusion of β-chitin fibers, which contribute to maintaining buoyancy by increasing the effective cell surface area [34,35]. In untreated *S. meneghinianus*, nanofibers can be observed under electron microscopy, with extrusion sites identified at both the valve margin and the central area (Figure 4A–F). Additionally, one or two rimoportulae are positioned near the valve margin, aligned with a costa, while marginal spines, when present, are also arranged along the costae. The density of costae (interstriae) ranges from 11 to 12 per 10 µm.

### 3.2. Effect of Pretreatment on the Purity and Microstructural Integrity of S. meneghinianus Biosilica

**Figure 5:**
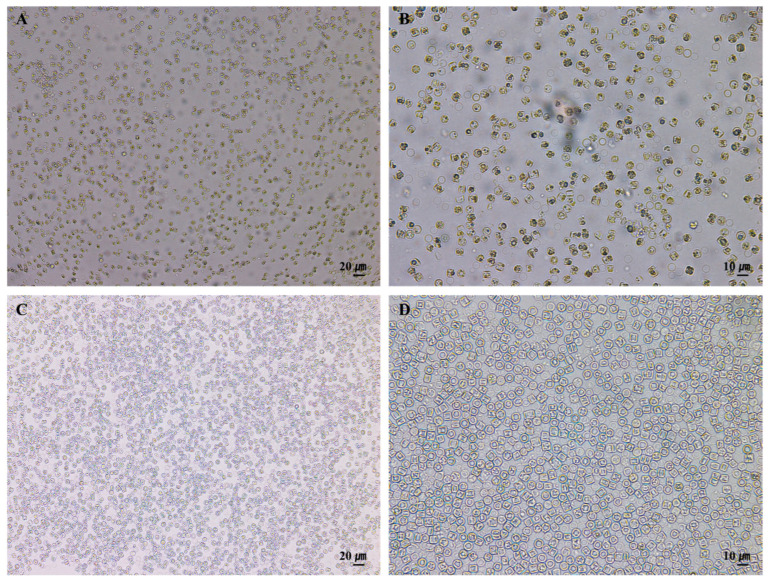
Pretreated *S. meneghinianus*. (**A**,**B**) Treated with hydrogen peroxide (H_2_O_2_) only. (**C**,**D**) Treated with a mixture of ethanol and hydrogen peroxide.

**Figure 6:**
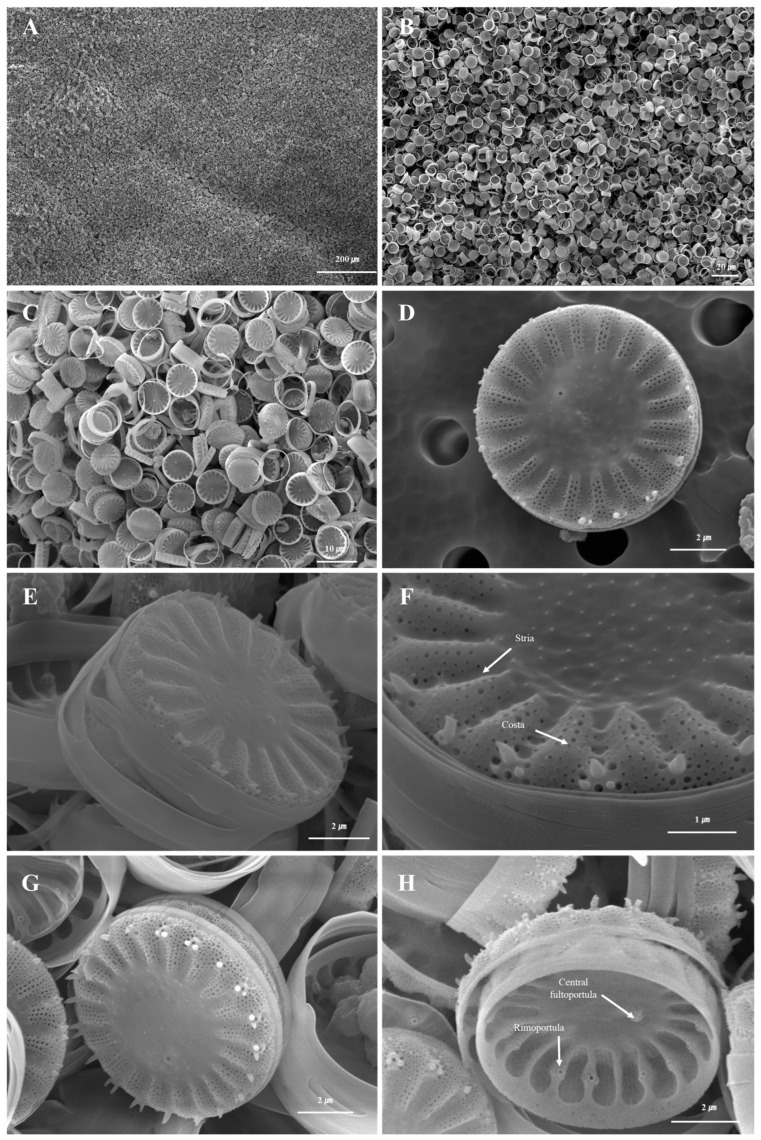
Microstructure of pretreated *S. meneghinianus*. (**A**–**C**) Overall morphology of pretreated *S. meneghinianus*. (**D**–**G**) Pretreated valves showing the absence of extracellular mucilage. (**H**) Internal structure of the valve. (Scale bar: (**A**) = 200 µm; (**B**) = 20 µm; (**C**) = 10 µm; (**D**,**E**,**G**,**H**) = 2 µm; (**F**) = 1 µm).

When only hydrogen peroxide was used without ethanol pretreatment, it was difficult to obtain pure biosilica from *S. meneghinianus* due to incomplete removal of chloroplast residues (Figure 5A,B). However, when ethanol treatment was applied prior to hydrogen peroxide treatment, pure white diatom frustules were successfully obtained (Figure 5C,D). Analysis of the microstructure of *S. meneghinianus* after pretreatment and purification confirmed the complete separation of the epitheca and hypotheca, as well as the effective removal of mucilage materials (Figure 6). Notably, despite undergoing pretreatment and purification processes, no morphological or structural damage was observed in the frustules of *S. meneghinianus*, including the preservation of striae, costae, fultoportulae, spines, and the porous architecture (Figure 6D–H). Moreover, the separation of the epitheca and hypotheca allowed for detailed observation of the internal structures, including the clear identification of rimoportulae and fultoportulae (Figure 6H). This separation is expected to provide a larger surface area for the loading of active substances, such as pharmaceuticals or cosmetic agents, thereby enhancing its potential as a functional biomaterial [12].

### 3.3. Assessment of Crystallinity and Elemental Composition in Pretreated S. meneghinianus

**Figure 7:**
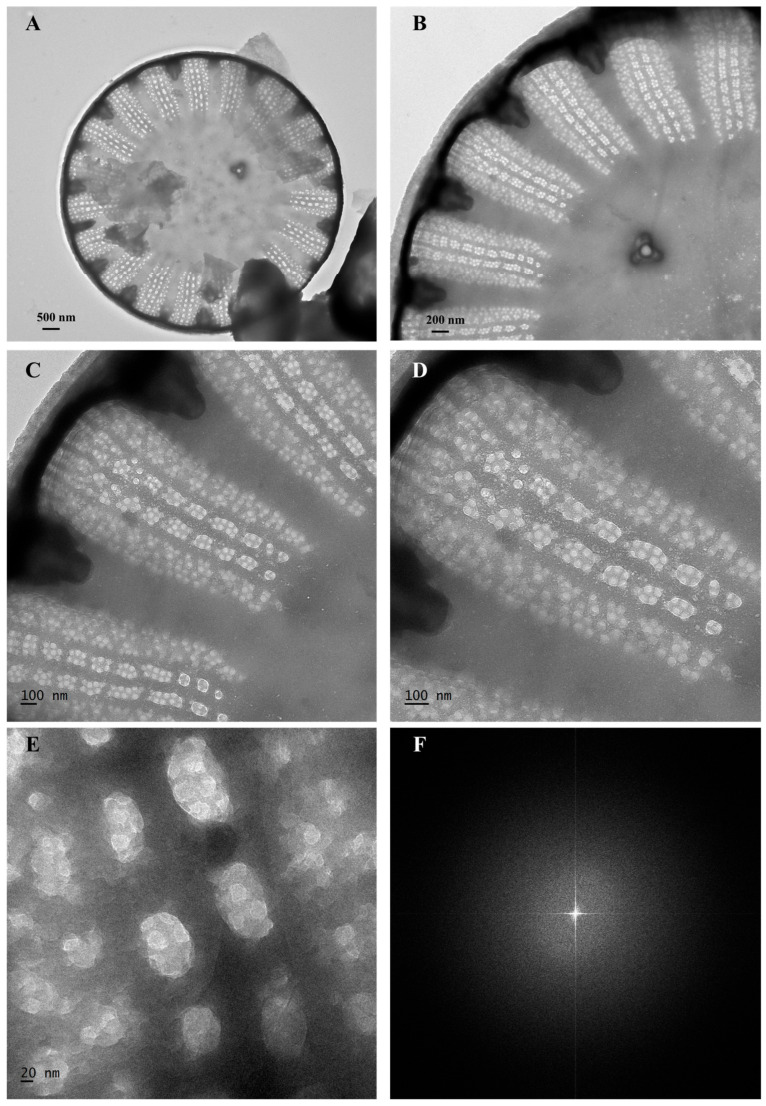
Porous pattern and FFT images of pretreated *S. meneghinianus* observed by transmission electron microscopy (TEM). (**A**) Valve of *S. meneghinianus*. (**B**–**E**) Porous patterns. (**F**) FFT image. (Scale bar: (**A**) = 500 µm; (**B**) = 200 µm; (**C**,**D**) = 100 µm; (**E**) = 20 µm).

The pore size and structure of *S. meneghinianus* biosilica were further examined using transmission electron microscopy (TEM). In the central region, the presence of fultoportula was observed, with a diameter of approximately 180 nm (Figure 7A,B). Although pores were visible under scanning electron microscopy (SEM), TEM analysis revealed that pores were absent in the striae and were instead concentrated along the costa (Figure 7A–E). The pores appeared to have an elongated oval shape (Figure 7A,B), and interestingly, smaller circular nanopores were found within these larger ovals (Figure 7C,D). The size of the larger oval pores ranged from 100 to 200 nm (Figure 7C,D), while the smaller circular pores measured approximately 20 nm in diameter (Figure 7E).

**Figure 8:**
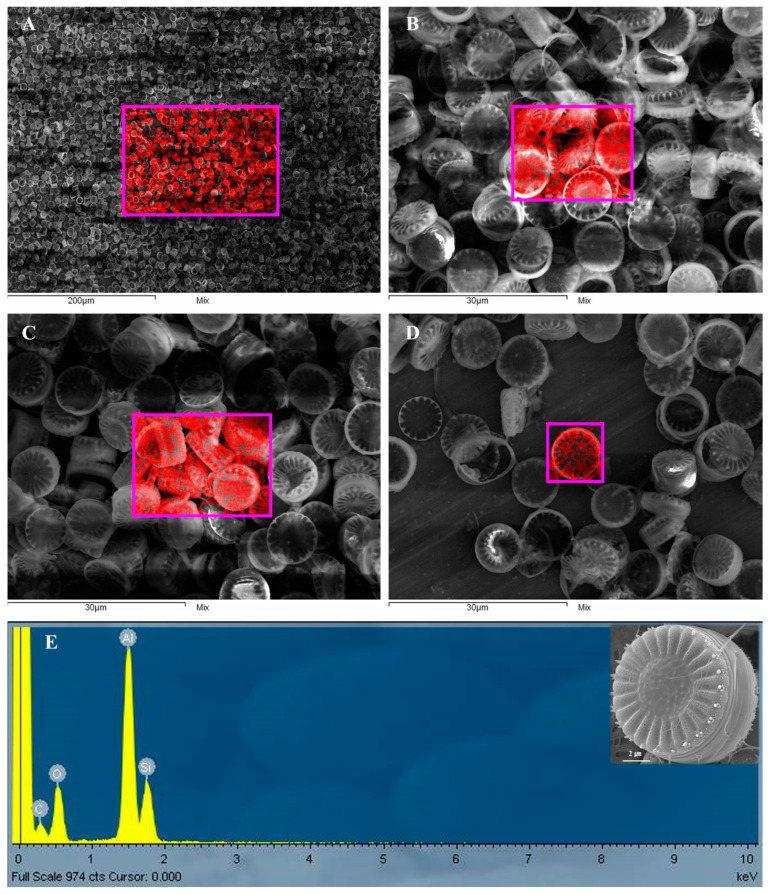
Elemental composition analysis of pretreated *S. meneghinianus.* (**A**–**D**) Mapping image showing the distribution of silicon (Si) in the pretreated *S. meneghinianus* frustules. (**E**) EDX spectrum of pretreated *S. meneghinianus* showing Si and other elements.

To indirectly assess the biodegradability of S. meneghinianus, fast Fourier transform (FFT) imaging was performed to evaluate its crystallinity. The analysis showed no distinct lattice patterns or Bragg diffraction rings; instead, scattered diffuse ring patterns typical of amorphous structures were observed (Figure 7F). This observation is consistent with previous reports on diatomaceous earth-derived amorphous silica, which also exhibited broad XRD peaks and diffuse diffraction patterns characteristic of amorphous materials [36]. Since biodegradability is often correlated with the absence of long-range structural ordering, these results suggest a potential for biodegradation under biological conditions. Such amorphous structures, characterized by randomly arranged polymer chains, are known to facilitate microbial or enzymatic access, thereby enhancing biodegradability [36,37]. Furthermore, energy dispersive X-ray spectroscopy (EDX) analysis confirmed that the primary element composing the pretreated *S. meneghinianus* was silicon (Si) (Figure 8A–D). A high content of aluminum (Al) was attributed to the aluminum sample stub, while the elevated carbon (C) signal resulted from the carbon tape used for mounting the samples during SEM analysis. The presence of oxygen (O) was expected due to the inherent SiO_2_ structure of the diatom biosilica (Figure 8E). Quantitative analysis of the EDX data indicated that the biosilica comprised 21.63 wt% silicon (17.24 at%), 31.07 wt% oxygen (43.50 at%), and 47.30 wt% aluminum (39.26 at%) (Table 3). In summary, the pretreatment process successfully yielded biosilica material predominantly composed of silicon and oxygen, with no remaining organic matrix, clear separation of the epivalve and hypovalve, and intact morphological structures. This establishes *S. meneghinianus* biosilica as a promising bioderived material for further applications.

### 3.4. Optimization of Culture Conditions for S. meneghinianus Growth

**Figure 9:**
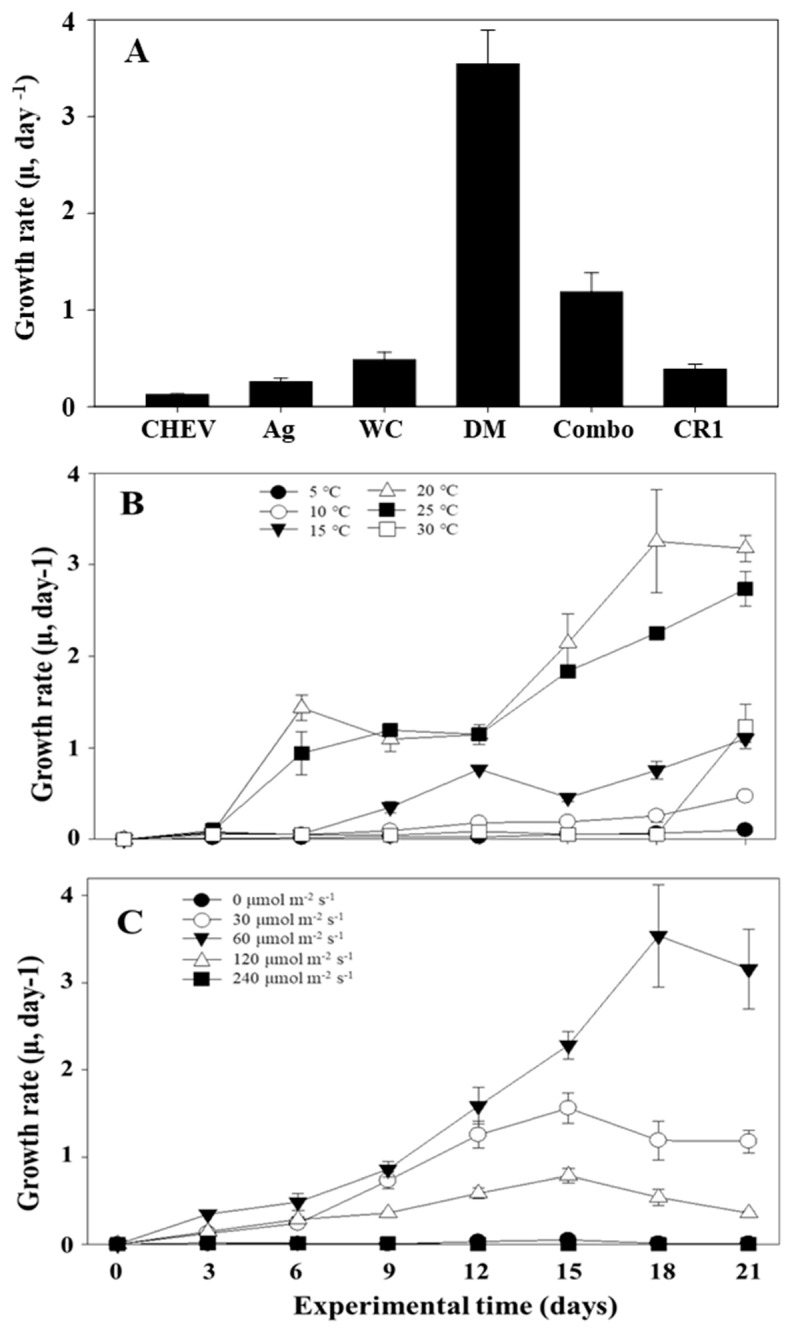
Growth rates of *S. meneghinianus* under various culture (**A**), temperature (**B**), and light conditions (**C**).

To determine the optimal growth conditions for *S. meneghinianus*, cell density was measured over time under different medium, temperature, and light intensity conditions with a constant initial inoculum density. Although DM medium is widely used for freshwater diatoms, several other media, including CHEV, AG, WC, Combo, and CR1, are also available for culturing freshwater species. Therefore, a preliminary test was conducted to identify the most suitable growth medium for *S. meneghinianus*. After three weeks of cultivation with identical initial inoculation densities, the specific growth rates were recorded as follows: CHEV (0.125 ± 0.008), AG (0.256 ± 0.038), CR1 (0.385 ± 0.055), WC (0.486 ± 0.078), Combo (1.186 ± 0.198), and DM (3.545 ± 0.348). Among these, DM medium supported the highest growth rate (Figure 9A). Based on this result, DM medium was selected for subsequent experiments assessing the effects of temperature and light intensity on growth. The temperature-dependent growth experiment was conducted within a range of 5 to 30 °C for three weeks, and significant differences in cell density were observed among the tested temperature conditions. The specific growth rate (μ, day^−1^) of *S. meneghinianus* under different temperature conditions is shown in Figure 9B. The growth rate remained negligible at 5 °C and 10 °C throughout the experimental period. At 15 °C, a slight increase was observed after day 9, but the growth remained relatively low compared to higher temperature treatments. The optimal growth was recorded at 25 °C, with the highest specific growth rate of approximately 3.2 day^−1^ on day 18. Growth at 20 °C was also favorable, reaching a maximum of about 2.3 day^−1^ on day 18. In contrast, although the initial growth at 30 °C was relatively fast, it plateaued after day 12 and did not exceed the growth observed at 25 °C (Figure 9B). As shown in Figure 9C, the specific growth rate of *S. meneghinianus* was significantly influenced by light intensity. No substantial growth was observed under dark conditions (0 μmol m^−2^ s^−1^) and at 30 μmol m^−2^ s^−1^. A gradual increase in growth rate occurred at 60 and 120 μmol m^−2^ s^−1^, with the latter reaching a peak value of approximately 2.6 day^−1^ on day 18. The highest growth rate was obtained under 240 μmol m^−2^ s^−1^, showing a continuous increase throughout the experimental period, peaking at around 3.2 day^−1^ on day 18. This result indicates that *S. meneghinianus* exhibits optimal growth at higher light intensities, particularly above 120 μmol m^−2^ s^−1^ (Figure 9C). Overall, the growth rate of *S. meneghinianus* was highest in the commonly used DM medium, and the species exhibited a preference for relatively low temperatures (20–25 °C) and moderate light intensity (60 μmol m^−2^ s^−1^). This growth pattern is consistent with previous reports in the literature [38,39].

## 4. Discussion

Approximately 19,688 species of diatoms have been reported worldwide, displaying remarkable morphological and size diversity [40]. Recently, the taxonomic classification of diatoms has been revised from *Bacillariophyta* to *Heterokontophyta*, which also includes classes such as *Phaeophyceae* and *Chrysophyceae* [40]. Diatoms are divided into four classes: *Bacillariophyceae* (15,350 species), *Mediophyceae* (2060 species), *Coscinodiscophyceae* (1867 species), and *Bacillariophyceae* incertae sedis (411 species). In Korea, a total of 2373 diatom species have been reported as of 2024, indicating a notably higher diversity compared to other microalgal groups [41]. The distinctive feature of diatoms lies in their highly intricate, nanoporous silica cell walls, known as valves. Each species exhibits unique pore patterns, which contribute to enhancing photosynthetic efficiency and enabling buoyancy and surface attachment in aquatic environments [42]. These structures are naturally formed through genetic and biochemical processes within the cell, resulting in precise and complex architectures that are challenging to replicate artificially [43,44]. This inherent self-assembly capability of biosilica offers significant potential for applications in material science and biotechnology [13,19]. Recently, the nanoporous architecture of diatoms has attracted considerable interest for its potential use in various fields such as drug delivery systems, biosensors, tissue engineering, and environmental remediation technologies [18,45]. Particularly, diatom-derived biosilica is known for its excellent biocompatibility and ease of surface modification, making it a promising candidate for developing functional nanomaterials [13,18,19]. In environmental and energy applications, diatom biosilica can be utilized as adsorbents and filtration materials for removing heavy metals and micropollutants, providing an eco-friendly, low-cost, and scalable alternative for sustainable material development [46]. Based on this background, the present study aimed to investigate the optimal growth conditions and microstructural characteristics of the freshwater diatom *S. meneghinianus*. Generally, diatoms exhibit favorable growth in low-temperature aquatic environments [47]. Consistent with previous reports, *S. meneghinianus* demonstrated its highest growth performance under low-temperature (20 °C) and low-light (60 μmol m^−2^ s^−1^) conditions. After three weeks of cultivation, the cell density increased by approximately 3.5-fold (Figure 9B,C). Although this growth rate is relatively lower compared to green algae and cyanobacteria, it aligns with the typical growth pattern observed in diatom species [48]. In the comparative growth experiment using different culture media, the highest growth rate of *S. meneghinianus* was observed in DM medium, corroborating the findings of previous studies on the optimal culture environments for freshwater diatoms [49]. Additionally, temperature and light condition experiments indicated that moderate light intensity and lower temperature conditions favored the growth of this species, reflecting its environmental adaptability [50,51]. These findings are consistent with existing literature [38,39]. From a materials science perspective, the precise and repetitive nanoporous structure of *S. meneghinianus* presents high potential as a biomimetic material. Its silica frustule offers a high surface area, robust mechanical strength, and tunable porosity, making it suitable for applications in catalysis, biosensing, nanophotonics, and drug delivery platforms [10,52]. In particular, the double-layered porous frustule of *S. meneghinianus*, characterized by highly organized hierarchical nanopore patterns, provides enhanced surface area and material transport properties compared to other freshwater diatom species. This distinctive morphology broadens its applicability in advanced functional materials, including controlled drug delivery systems, optical coatings, and catalytic nanostructures [12,15]. Recent research has also explored its potential as a natural template for the fabrication of metal oxide nanostructures through surface modification and deposition techniques [18,53]. Biosilica, produced through biological processes in diatoms, is formed under low temperature and low energy conditions, resulting in highly species specific and sophisticated structures that are difficult to reproduce through conventional synthetic silica processes [54]. Its combination of natural precision and chemical versatility highlights its potential as a sustainable, bio-based material capable of addressing limitations in existing silica-based materials [12,15]. This study provides valuable insights into the growth optimization and ultrastructural characteristics of the freshwater diatom *S. meneghinianus*, which has been relatively underexplored in both domestic and international research. The secured biosilica resources and structural analyses presented here are expected to serve as fundamental data for future research in bio-based materials, environmental applications, and energy technologies. Further studies focusing on the development of improved culture systems, optimized growth conditions, and large scale biosilica production and materialization will likely expand the application potential of this species across diverse fields. Moreover, the successful establishment of a long-term monoculture system under optimized freshwater conditions in this study demonstrated the feasibility of producing high-purity biosilica with preserved structural integrity. These results not only address the limitations of marine diatom-based biosilica production but also position *S. meneghinianus* as a sustainable and eco-friendly alternative source for nanostructured biosilica materials applicable to biomedical, environmental, and energy sectors. Further studies focusing on functional performance evaluation, such as adsorption efficiency and biocompatibility tests, are planned based on the biosilica resources secured in this study.

## 5. Conclusions

This study comprehensively characterized the morphology, microstructure, and growth properties of the freshwater centric diatom *S. meneghinianus* and evaluated an effective pretreatment method for obtaining high purity biosilica. The isolated *S. meneghinianus* exhibited relatively small and uniform cell sizes ranging from 8 to 10 µm, with distinct morphological features such as regularly arranged spines, costae, striae, and both central and marginal fultoportulae. Electron microscopy confirmed the presence of highly ordered nanoscale pores predominantly distributed along the costae, with approximately 4000 pores per valve.

The pretreatment method incorporating ethanol and hydrogen peroxide proved highly effective in removing organic residues and mucilage, yielding intact and structurally preserved biosilica frustules. Crystallinity analysis via fast Fourier transform (FFT) revealed the amorphous nature of the biosilica, suggesting favorable biodegradability. Energy dispersive X-ray spectroscopy (EDX) confirmed that the purified frustules were primarily composed of silicon and oxygen, with no detectable organic matrix.

Optimization experiments for growth conditions demonstrated that *S. meneghinianus* achieved the highest specific growth rate in DM medium, particularly at temperatures of 20–25 °C and light intensities of 60–120 μmol m^−2^ s^−1^. This growth pattern is consistent with known ecological preferences of freshwater diatoms and establishes a reliable cultivation protocol for large scale biomass production.

Collectively, these findings highlight *S. meneghinianus* as a promising bioresource for sustainable biosilica production. The high purity, structural integrity, and biodegradability of its biosilica, combined with its efficient growth under optimized conditions, suggest strong potential for applications in biomaterials, nanotechnology, pharmaceuticals, and environmental remediation. Future studies should focus on the functionalization and application specific performance of *S. meneghinianus*-derived biosilica in advanced material systems.

## Figures and Tables

**Table 1:** Information on sampling sites.

Site	Latitude (N)	Longitude (E)	Temperature (°C)	DO (mg/L)	pH	Sample Species
AD	37°33′38.69″	128°44′37.11″	12.7	10.13	7.79	*Stephanocyclus meneghinanus*

**Table 2:** Duplicated experiments different temperature, light intensity, pH, and nutrient conditions.

	Temperature (°C)	pH	Medium	Light Intensity (μmol/m^2^·s)	Analysis
Exp. 1	20	7	CHEV Ag WC DM Combo CR1	60	Growth rate (μ, day^−1^)
Exp. 2	5 10 15 20 25 30	7	DM medium	60	
Exp. 3	20	7	DM medium	0 5 15 30 50 100	

**Table 3:** EDX-derived elemental composition (wt% and at%) of pretreated *S. meneghinianus* biosilica.

Element	Weight (%)	Atomic (%)
O	31.07	45.50
Al	47.30	39.26
Si	21.63	17.24

## Data Availability

Data is contained within the article.

## Abbreviations

The following abbreviations are used in this manuscript:

SEM	Scanning Electron Microscopy
TEM	Transmission Electron Microscopy
EDX	Energy Dispersive X-Ray Spectroscopy
AD	Andong Dam
